# The stressed eyewitness: the interaction of thematic arousal and post-event stress in memory for central and peripheral event information

**DOI:** 10.3389/fnint.2012.00057

**Published:** 2012-08-23

**Authors:** Gerald Echterhoff, Oliver T. Wolf

**Affiliations:** ^1^Social Psychology Group, Department of Psychology, University of MünsterMünster, Germany; ^2^Cognitive Psychology Group, Department of Psychology, Ruhr-University BochumBochum, Germany

**Keywords:** eyewitness memory, stress, arousal, salivary cortisol, social influence

## Abstract

Both arousal during the encoding of stimuli and subsequent stress can affect memory, often by increasing memory for important or central information. We explored whether event-based (thematic) arousal and post-event stress interact to selectively enhance eyewitnesses' memory for the central aspects of an observed incident. Specifically, we argue that memory for stimuli should be enhanced when (1) the stimuli are encoded under arousal (vs. non-arousal), and (2) stress is experienced soon after the encoding episode. We designed an experiment that extended previous research by manipulating arousal without changing the stimulus material, distinguishing between central and peripheral event information, and using a dynamic, life-like event instead of static pictures. After watching a video depicting a burglary under high or low thematic arousal, psychosocial stress was induced or not induced by the Trier Social Stress Test (TSST). Salivary cortisol was measured at standard intervals. Consistent with our prediction, we found a significant post-event stress × thematic arousal × centrality interaction, indicating that the recognition advantage for central event items over peripheral event items was most pronounced under both high thematic arousal and post-event stress. Because stress was induced after encoding this interaction cannot be explained by possible differences at encoding, such as narrowed attention. The centrality effect of post-event stress under high thematic arousal was statistically mediated by the cortisol increase, which suggests a key role of the stress hormone. We discuss implications of our findings for psychological and neuroscientific theories of emotional memory formation.

## Introduction

Imagine the following scene: from your window you see someone moving around in the neighbor's house in a suspicious manner. Soon, you feel confident that you are witnessing a burglary in the middle of the day. You know that your neighbors have just returned early from their vacation and are taking an afternoon nap upstairs. Envisioning a possibly harmful encounter between the burglar and your neighbors you start feeling agitated. After watching in petrified anticipation for a little while, you call the police. The burglar has disappeared when the police arrive. A police officer immediately starts a brief interrogation, asking you who you are and what you were doing. You cannot help feeling being treated like a suspect. You feel stressed and your heart is beating. While you wait for more questions, you meet another witness who retells the incident in some detail. Finally, after you have calmed down, you try to remember the incident, including central aspects (e.g., items carried by the burglar) but also peripheral details (e.g., items that remained untouched).

This episode illustrates the questions we address in our research: How is one's memory for an event, specifically memory for central and peripheral information, affected by arousal during the encoding of the event (in our example, the witnessing of the burglary), post-event stress (the first, stressful inter-rogation), and additional post-event information (the other witness's retelling)? Laboratory research over the last decades has started to characterize the effects of emotional arousal and stress on memory. Arousing material is typically better remembered than neutral material, an effect mediated by the sympathetic nervous system (SNS) and its release of the catecholamines adrenalin and noradrenalin (see Cahill and McGaugh, 1998). The emotional memory enhancement appears to be especially pronounced for central aspects of the arousing item, whereas emotional arousal often impairs memory for peripheral details. This has been interpreted as a result of attentional narrowing (Easterbrook, 1959; Christianson, 1992). Post-encoding effects mediated via a modulation of to be consolidated information might also contribute to this effect.

Arousal is associated with an activation of the SNS, a more serious threat to the physical or social self in contrast leads to stress, accompanied by activation of the hypothalamus pituitary adrenal (HPA) axis (Mason, 1968; Dickerson and Kemeny, 2004). The hormones of the HPA axis, namely Corticotrophin Releasing Hormone (CRH), Adrenocorticotrophin (ACTH), and cortisol are known to influence learning and memory. In real life, as illustrated by our example, arousal and stress may occur in short succession. Thus, an important research question is how stress interacts with arousal to influence memory.

The influence of post-learning stress on memory has been investigated by Cahill et al. (2003). The authors employed pictures that evoked different levels of arousal. It was found that post-learning stress selectively enhanced memory for the arousing slides. Hence, in this study memory depended on the interaction between post-learning stress and the level of arousal during encoding. Similar findings have been reported by others (e.g., Smeets et al., 2008). This observation is also consistent with findings from animal studies indicating that noradrenergic arousal is a prerequisite for the modulatory effects of cortisol or other glucocorticoids on memory (for a review, see Roozendaal et al., 2006).

Although some studies did not find an interaction between arousal and the effects of stress or glucocorticoid manipulations on memory (e.g., Preuss and Wolf, 2009), the above findings suggest a possible interaction between arousal and post-encoding stress: memory for stimuli is enhanced when (1) the stimuli are encoded under arousal (vs. non-arousal), and (2) stress is experienced soon after the encoding episode.

However, extant research faces three main limitations. First, existing manipulations of emotion or arousal have been particularly afflicted by the problem of potential confounds to the extent that they have relied on different stimulus material (negative or arousing vs. neutral). Stimulus-based variations in arousal lead to unavoidable confounds between arousing vs. neutral pictures. Effects for emotionally arousing visual stimuli, such as the sight of a wound or a weapon, may be due to attentional capturing, visual salience, or novelty rather than arousal *per se* (see Mather and Sutherland, 2011). An alternative approach is the use of thematic arousal, for instance, arousal induced by an accompanying story or by the instructions given to the subjects (Laney et al., 2004; Payne et al., 2007; also see Heuer and Reisberg, 1990; Cahill and McGaugh, 1995). A manipulation of thematic arousal induces different experiences of the same stimulus material, thus circumventing the problems of stimulus-based manipulations. Studies that have manipulated thematic arousal, however, have not examined the interplay between thematic arousal and post-learning stress.

Second, most research on emotional memory has used static pictures with different emotional contents as stimulus material, for instance, from the International Affective Picture System (IAPS) data base. While this approach has several advantages, real-life events are dynamic rather than static. Hence, the focus on static pictures constrains generality and external validity of the research. Beckner and coworkers (2006) have used a movie in order to assess the impact of post-learning stress on memory. They found, in line with Cahill et al. (2003), enhancing effects of post-event stress on memory. However, those authors did not vary the level of arousal induced by the movie and reported that the movie about a dinner party was not intended to be arousing (Beckner et al., 2006). In the domain of military survival training, Morgan and colleagues (2004) tested the accuracy of eyewitness identification of military personnel who had interrogated the participants under either extremely high or low stress. Overall, identification accuracy was better under low stress. However, the study differed in several respects from the present approach, mainly because the source of stress and the to-be-remembered stimulus were confounded, there was no separate induction of arousal, and stress occurred already during the encoding phase.

Third, previous studies of post-event stress effects on memory have not distinguished between central and peripheral details (Cahill et al., 2003; Rimmele et al., 2003). However, classical research has found that memory for peripheral and central information can be differentially affected by arousal (Christianson, 1992). Studies on patients with amygdala damage suggest that the effect of emotional arousal on memory for central (vs. peripheral) material is subserved by the amygdala (Adolphs et al., 2005). It has been found that glucocorticoids released during stress influence memory consolidation via a modulatory effect on the amygdala (Cahill and McGaugh, 1998). Hence, effects of post-event stress might differ for central versus peripheral details of an arousing event.

The goal of the present study was to investigate how post-encoding stress will affect memory depending on the thematic arousal of the initial learning episode. In so doing, we wanted to redress the shortcomings described above. Specifically, we examined whether this effect differs for peripheral and central details of the witnessed event. Drawing on the research discussed above we predicted that under high thematic arousal post-learning stress would enhance memory for the central elements of an event (e.g., a cashbox grabbed by the burglar) at the expense of peripheral items (e.g., a video tape remaining untouched on a shelf). We also examined whether the increase in cortisol would statistically mediate such a potential stress effect. To our knowledge, such an analysis has not been reported.

Furthermore, we explored possible effects on memory for false post-event information. Published research on the role of stress in experimentally induced false memories is scarce. The few extant studies (Payne et al., 2002; Smeets et al., 2006, 2008) have focused on false memories for word stimuli resulting from semantic associations in the Deese-Roediger-McDermott paradigm (Roediger and McDermott, 1995), yielding mixed evidence. In the present study, we examined whether thematic arousal during observation and post-event stress would moderate the extent to which participants would falsely remember post-event misinformation. Participants read a post-event narrative about the witnessed event that contained several false details. These false details were minor additions to the actual scenes (e.g., a tennis racket on a basement shelf in the background) and thus were more similar to peripheral (vs. central) information. Impaired memory for peripheral information resulting from stress or arousal (e.g., Christianson, 1992) could thus facilitate the implantation of false details, leading to a greater effect of post-event misinformation on eyewitness memory. On the other hand, it has been found that negative mood reduces the effect of post-event misinformation, presumably due to enhanced, and more “suspicious,” bottom-up scrutiny of the environment (Forgas et al., 2005).

Because the event description predominantly reported correct information, it also allowed participants to rehearse, or re-encode, the described parts of the witnessed event. Hence, we also examined the effect of this rehearsal on memory for correct event details.

## Materials and methods

### Overview

**Figure 1:**
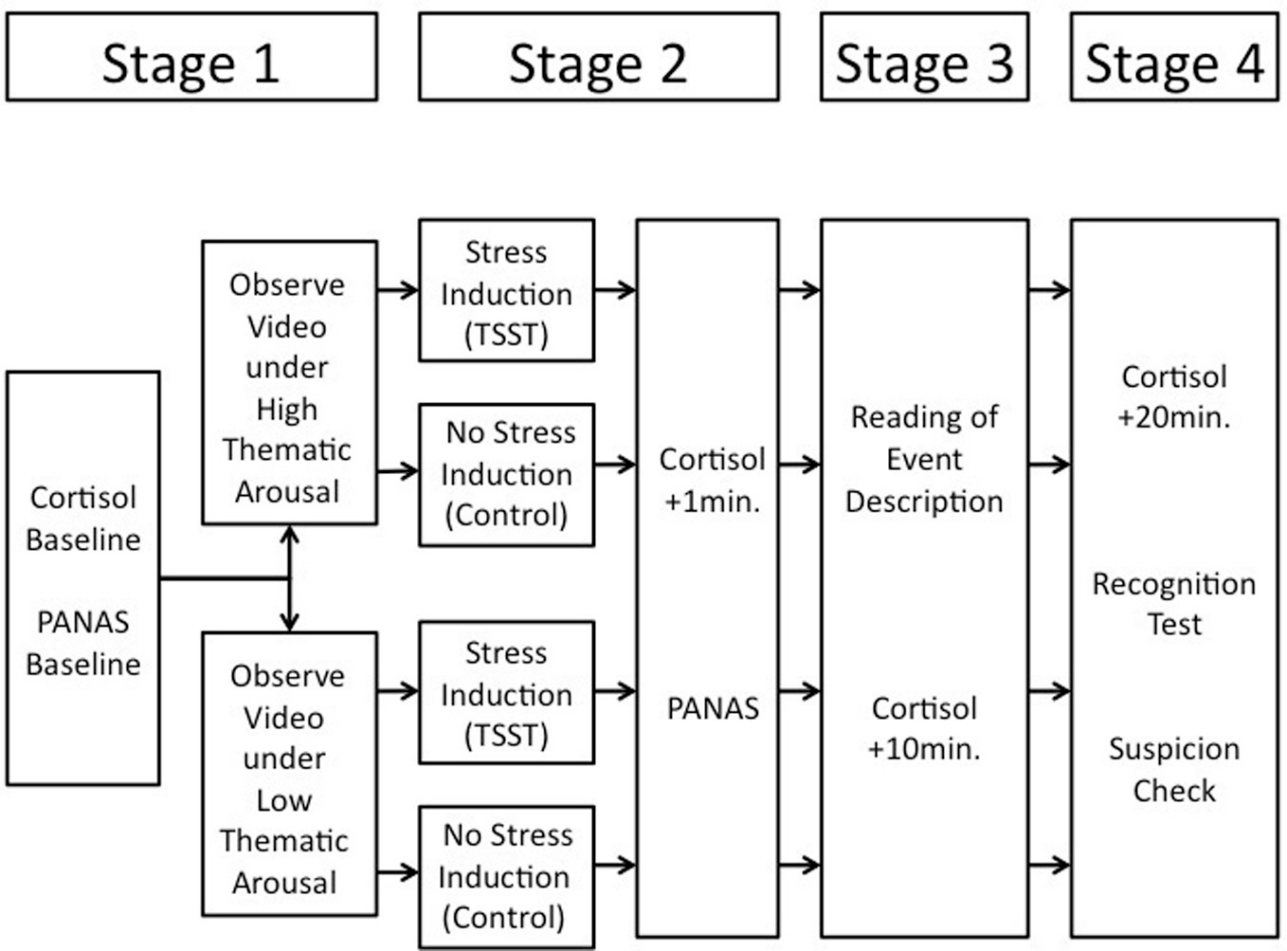
**Overview of the procedure; the duration of the video was ~12 min, the duration of the TSST was ~15 min**.

The experiment consisted of four main stages (see Figure 1), (1) encoding of a target event (with or without thematic emotional arousal), (2) manipulation of psychosocial stress, (3) rehearsal of the event information based on an event description, which contained many correct event details and some additional false, non-event details, and (4) a recognition test for the target event.

In the first stage, participants witnessed a video-filmed event depicting a burglary. Arousal during the viewing of the event (low vs. high arousal) and subsequent stress (post-event stress vs. no-post-event stress control) were varied independently. In the high thematic arousal condition, participants received instructions that were designed to produce heightened emotional arousal during the encoding of the target event. Specifically, participants were induced to anticipate seeing a version of the video showing a distressing, possibly violent incident. In the low thematic arousal condition, participants learned that the event they would see was unlikely to be experienced as distressing. This manipulation induces different emotional arousal concerning the *same* content material. Thus, it avoids potential confounds of manipulations inducing different levels of arousal by presenting participants with to-be-remembered material that is either emotionally arousing (for instance, shocking pictures) or neutral (see Cahill and McGaugh, 1995; Laney et al., 2004).

In the second stage, after event encoding, a stress manipulation was employed. For approximately half of the participants from both thematic arousal groups we administered the Trier Social Stress Test (TSST; Kirschbaum et al., 1993) to induce psychosocial stress (*post-event stress* condition). The remaining participants experienced a non-stressful situation (*no-stress control* condition). In the third stage, after the stress manipulation, participants were asked to read a narrative description of the witnessed incident. No further reason was given for including this description. For the most part, the description correctly described the original event but also contained some non-event items (i.e., items not shown in the original event).

After a short interval, we administered a yes/no recognition test. The test contained event items (i.e., items that did appear in the video-filmed event) and non-event items (i.e., items that did not appear in the video-filmed event). Based on previous research employing the same material (Echterhoff et al., 2007), we distinguished between peripheral and central event items. Among the non-event items, half were items that were falsely mentioned in the event description (false additional items), the other half were items that appeared in neither the video-filmed event nor the narrative (new items). Cortisol measures were taken at the beginning of the test session (baseline), and then approximately 1, 10, and 20 min after the TSST.

### Participants

Participants were 88 male students at Bielefeld University (mean age 24.3 years, ranging from 19 to 37). They were informed that the study was about the perception and communication of events. None of the participants reported suffering from acute or chronic diseases or taking medication. The data were collected in two main waves, with the first wave (*n* = 43) taking place one semester before the second wave (*n* = 45). Each participant received either a compensation of 10 € or curricular credit. The experiment, including the treatment of human subjects, was approved by the ethics committee of the German Psychological Association (Deutsche Gesellschaft für Psychologie). The guidelines of the Declaration of Helsinki and standards of the American Psychological Association (APA) were followed. Informed consent was obtained from all participants.

### Design

The basic design of the experiment was 2 (low vs. high thematic arousal) × 2 (post-event stress vs. no post-event stress), varied between participants. For analyses of event items, we also included centrality (central vs. peripheral) and rehearsal (rehearsed vs. non-rehearsed) as two within-participants factors, yielding a 2 × 2 × 2 × 2 mixed design. For analyses of non-event items, addition in the event description (false additional vs. new) was employed as a within-participants factor, yielding a 2 × 2 × 2 mixed design. The primary dependent variable was the proportion of “yes” responses in the recognition test, analyzed separately for the different levels of the within-participants factors (i.e., the different item types).

### Materials

As the target event we used a video (lasting ~12 min) that had been employed successfully in previous studies of eyewitness memory (Echterhoff et al., 2005, Experiment 4; Echterhoff et al., 2007). The video depicted a burglar searching a house for valuables after a resident (a young woman) has left. Following Echterhoff et al. (2007), we distinguished between central or peripheral event items. *Central event items* were clearly visible for an extended period of time, often at the center of the video image, and were manipulated by a protagonist (e.g., a cashbox grabbed by the burglar). In contrast, *peripheral event items* were presented for a shorter time, mostly at the periphery of the video image, and were not visibly manipulated by a protagonist (e.g., a video tape on a shelf in the living room). In the present study we employed 16 central and 16 peripheral event items. In previous pretests of the stimulus material, which did not include post-event information (Echterhoff et al., 2007), correct recognition for the central event items was high, without reaching a ceiling (hit rates between 0.80 and 0.90), whereas correct recognition for the peripheral event items was significantly lower, without reaching a floor (hit rates between 0.25 and 0.50). Thus, central event items were better remembered than were peripheral event items.

In the *high thematic arousal* condition participants received the following instruction just before watching the target video: “You will be watching one of two different versions of the video. In one of the two versions there is a surprising turn, involving a physical confrontation between the protagonists. In the other version there will be no such a confrontation. Although the version is selected by chance, it is more likely that you will see the version depicting the confrontation than the other version.” The expression “the protagonists” obviously referred to the young woman and the burglar. In the *low thematic arousal* condition, participants were just told that they would be watching one of two different versions of the video, and that these versions would differ in some visual features. A possible confrontation was not mentioned. In truth, there was no violent version of the video—the same (non-violent) version of the video was presented to all participants. We designed this type of manipulation to ensure that differences in participants' emotional states were not due to differences in the observed stimulus material but to differences in their expectation of possibly seeing arousing material. Thus, we could avoid confounds which are faced by arousal manipulations based on different content of the stimulus material, for instance shocking, unfamiliar, or perceptually salient vs. non-shocking, familiar, or non-salient (see Laney et al., 2004).

The effectiveness of this arousal manipulation was established in a pretest with 50 male participants (students at Bielefeld University, mean age 26.7 years) who received a compensation of 3 €. The pretest participants were randomly assigned to the high-arousal instruction condition (*n* = 25) or the low-arousal instruction condition (*n* = 25). Participants' arousal was assessed at two times: (1) midway through the video (after approximately half of the running time), and (2) immediately after the end of the video. At each time, participants completed four rating items on eight-point scales, each anchored with 1 (*not at all*) and 8 (*very much*): *How nervous do you feel? How tense do you feel? How calm are you right now? How relaxed are you?* (The latter two items were reverse coded.) To permit participants to provide their ratings at time 1 (i.e., in the middle of the video), the video was briefly interrupted. We did not probe for arousal by this procedure in the main experiment because the interruption could interfere with relevant memory processes such as the encoding of the target event. The reliability of the eight ratings (i.e., the four items administered during and after the video) was high (Cronbach's α = 0.92). The eight single scores were averaged, yielding one mean score of arousal for each participant. Arousal was significantly higher for participants in the high-arousal instruction (*M* = 3.24, SD = 1.42) than for participants in the low-arousal instruction condition (*M* = 2.65, SD = 1.24), *t*_(48)_ = 2.03, *p* = 0.048, η^2^ = 0.08 (two-tailed test).

As in previous studies (Echterhoff et al., 2005, Experiment 4; Echterhoff et al., 2007) we employed two different versions of the event description (each approximately 1000 words). Version A contained one half of the 32 event items, while version B contained the other half of the event items. Event items that were included in an event description were rehearsed (*rehearsed event items*), whereas event items that were not included in the event description were not rehearsed (*non-rehearsed event items*). The sets of 16 rehearsed and 16 non-rehearsed event items each consisted of eight central and eight peripheral items. Thus, the event description contained an equal number of central event items (e.g., the cashbox grabbed by the burglar) and peripheral event items (e.g., the video tape on a living-room shelf).

The event description also contained *non-event items*, i.e., items that were not shown in the target event (e.g., a tennis racket on a basement shelf). We used the same pool of 32 non-event items used in previous studies (Echterhoff et al., 2005, Experiment 4; Echterhoff et al., 2007). Version A of the event description contained one half (i.e., 16) of the non-event items, while version B contained the other half (i.e., 16) of the non-event items. Non-event items included in a description are called *false additional items*, whereas non-event items not included in a description are called *new items*. (New items were thus not presented in either the target event or the event description.) In previous pretests of the stimulus material, which did no employ post-event information (Echterhoff et al., 2007), false alarm rates for non-event items were significantly above 0 (between 0.15 and 0.25).

In the yes/no recognition test, participants decided whether items had appeared in the original event. The test consisted of 32 event items (eight non-rehearsed peripheral event items, eight rehearsed peripheral event items, eight non-rehearsed central event items, eight rehearsed central event items) and 32 non-event items (16 false additional items and 16 new items).

### Salivary collection and analysis

Saliva was collected using Salivette collection devices (Sarstedt, Nuembrecht, Germany). Samples were kept in a freezer until completion of the study. Salivary cortisol was measured out of these samples using a commercially available Immuno-Assay (IBL, Hamburg). Inter- and intra-variations of this assay are below 10%. The analyses were conducted in a biochemical laboratory under direction of Professor Clemens Kirschbaum at the Technical University of Dresden, Germany.

### Procedure

#### Stage 1

Upon entering the lab, participants read and signed an informed consent form that described the procedure of the study. To obtain a baseline measure of cortisol (referred to as cortisol baseline) a first saliva sample was taken from participants. Participants also completed the Positive and Negative Affect Schedule (PANAS; Watson et al., 1988). The PANAS consists of 20 adjectives describing negative (e.g., *angry*, *irritated*) and positive affective states (e.g., *attentive*, *excited*). The participants were asked to indicate how much the words matched their current mood (on rating scales from 0 to 5, with higher numbers indicating stronger agreement). Positive and negative affect measures can be independent, and related studies have typically found effects of stress on negative affect (see Schoofs et al., 2008); hence we focus on the analysis of negative affect. The mean score of the negative affect items from the PANAS scale from this initial administration served as a baseline measure.

Participants then watched the video depicting the target event on a TV monitor with a screen diagonal of 60 cm (24″) at a distance of approximately 1.50 m (5 ft.) either under low or high arousal. The low thematic arousal condition was employed in the first wave of the study, while the high thematic arousal condition was employed in the second wave of the study.

#### Stage 2

Next, post-event stress was manipulated. In the *post-event stress* condition (*n* = 44), we induced psychosocial stress with the TSST (Kirschbaum et al., 1993). The TSST consists of a short preparation period (2 min) followed by a 5-min self-produced speech (i.e., a speech in a fictitious job interview focusing on personal strengths and weaknesses) in front of a committee (consisting of a female and male confederate wearing white coats) and a 5-min mental arithmetic exercise (counting backwards from 2043 in steps of 17). During these procedures, participants are video-taped and can see themselves on a monitor in the back of the room. The TSST has been shown to reliably induce a significant activation of the HPA axis and the SNS (Kirschbaum et al., 1993). In the *no-post-event-stress* condition (*n* = 44), participants were asked to give a 5-min speech about a movie or a book of their choice and to perform mental arithmetic in an empty room for another 5 min (see Het et al., 2009). This control condition is relatively similar in physical and mental workload but lacks the stress-inducing components of the TSST, which are social evaluative threat and uncontrollability (Mason, 1968). In a recent meta-analysis, the TSST was found to provoke the most robust physiological stress responses (i.e., cortisol stress responses) relative to various other laboratory stress tasks (Dickerson and Kemeny, 2004).

Approximately 1 min after the TSST, a second saliva sample (cortisol +1 min) was taken. Participants completed again the PANAS (post-stress induction measure). As for the baseline measure, the mean negative affect score served as a subjective measure of the impact of post-event stress on participants' mood.

#### Stage 3

All following materials were presented on a Laptop with a 15-inch monitor using the experimental software MediaLab (Jarvis, 2005). Participants read the *event description*, which contained one of the two sets of 16 event items (eight central, eight peripheral) and one of the two sets of 16 non-event items, depending on the version of the description (A or B). We counterbalanced rehearsed and non-rehearsed event items as well as false additional versus new items by providing one half of the participants with version A of the event description and the other half of the participants with version B. An equal distribution of the two versions was ensured within the between-participants conditions.

After the presentation of the event description, ~10 min after the post-event stress manipulation, a third saliva sample (cortisol +10 min) was taken. We then administered two rating scales, which served as a filler task (lasting ~10 min).

#### Stage 4

Approximately 20 min after the post-event stress manipulation a fourth saliva sample (cortisol +20 min) was collected. Immediately afterwards, the yes/no recognition test was administered (for the items, see “*Materials*”). The items were presented in a random order and remained on the computer screen until participants responded by pressing a *yes* or *no* button. In a funneled post-experimental suspicion check, participants were first asked to guess the purpose of the study and then probed more specifically about their beliefs concerning the role of the TSST and the post-event description. The data of four participants were excluded from the analyses because the participants exhibited high insight into the rationale of the study, resulting in the sample described above (see “*Participants*”). In the debriefing session great care was taken to reduce the likelihood of negative consequences for participants in the post-event stress condition (see, e.g., Het et al., 2009).

All statistical tests were two-tailed, except when noted otherwise. Regarding effect size, we report η^2^ (eta squared) and η^2^_p_ (partial eta squared).

## Results

### Induction of post-event stress

**Figure 2:**
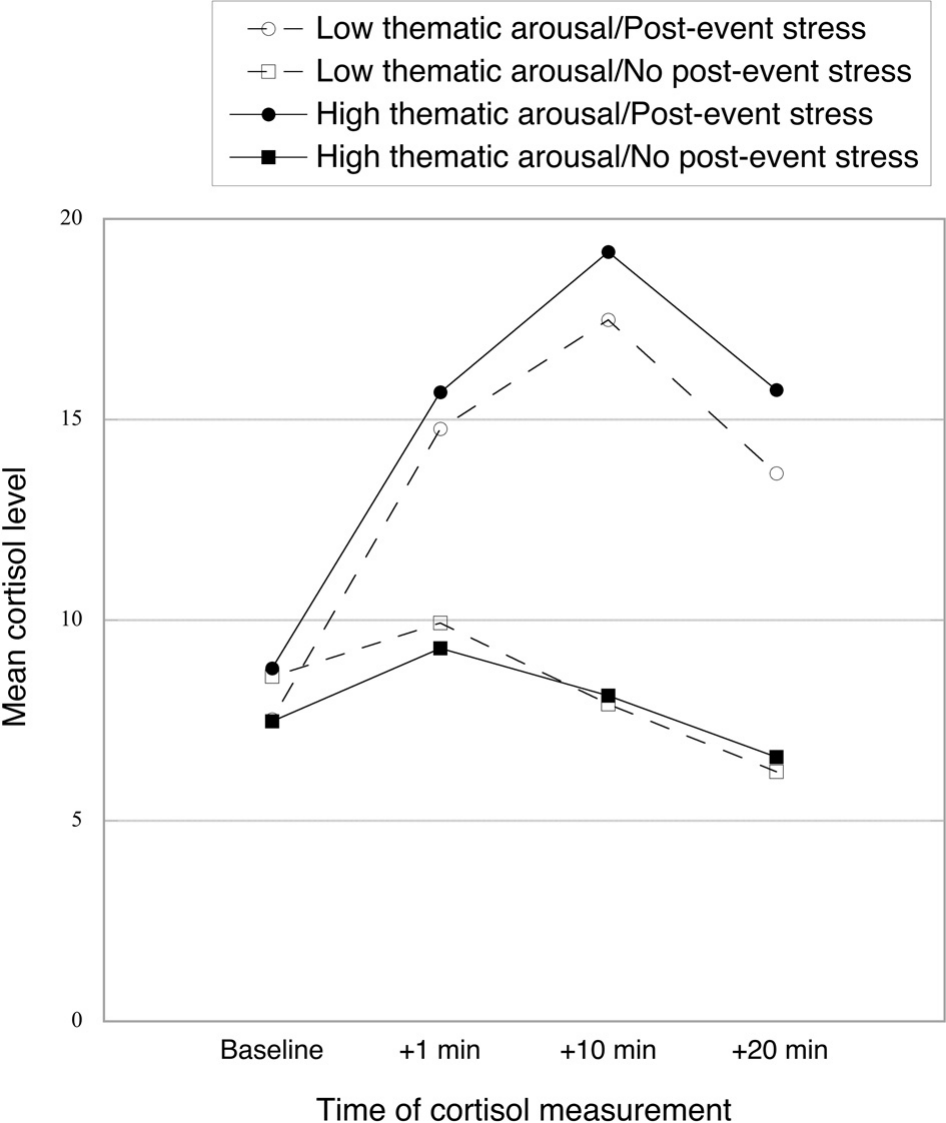
**Mean cortisol level (baseline and at 1, 10, and 20 min after the post-event stress manipulation) as a function of thematic arousal and post-event stress**.

As can be seen in Figure 2, the TSST successfully induced post-event stress at all three measurement times (+1, +10, and +20 min). We conducted a mixed 2 (post-event stress vs. no post-event stress) × 2 (low vs. high thematic arousal) × 4 (baseline vs. +1 vs. +10 vs. +20) ANOVA, with the first two variables varying between subjects and time of measurement varying within subjects. Cortisol concentrations were overall higher in the post-event-stress group (vs. the no-post-event-stress group), as indicated by a main effect of post-event stress, *F*_(1, 84)_ = 30.67, *p* < 0.001, η^2^_p_ = 0.27. Importantly, a significant main effect of measurement time [*F*_(3, 252)_ = 42.76, *p* < 0.001, η^2^_p_ = 0.34] was qualified by a significant two-way interaction between post-event stress and measurement time [*F*_(3, 258)_ = 40.83, *p* < 0.001, η^2^_p_ = 0.33], reflecting the larger increase in cortisol in the post-event-stress (vs. no-post-event-stress) condition. No other significant effects emerged, all *F*s < 1, *ns*. We also tested effects of post-event stress at each measurement time with pairwise comparisons. Whereas no effect of post-event stress was found for cortisol baseline (*F* < 1, *ns*), cortisol was significantly higher for the post-event-stress group than the no-post-event-stress group for other measurement times, *F*_(1, 84)_ = 14.28, 54.78, and 53.94; all *p*s < 0.001, η^2^_p_ = 0.15, 0.40, and 0.39; for cortisol +1, +10, and +20, respectively.

Within the post-event stress condition alone, all three cortisol measures after the TSST were significantly higher than the baseline measure, all *t*s_(43)_ > 7.85, all *p*s < 0.001 (calculated by pairwise comparisons). In this condition, the peak cortisol level was reached at +10 min; this level was the only one differing significantly from all three other levels, all *t*s_(43)_ > 4.25, all *p*s < 0.001. We calculated cortisol increase for use in subsequent analyses by subtracting the baseline scores from the mean of the three post-treatment cortisol measures. Existing research suggests that the thematic arousal manipulation would not induce changes in cortisol concentrations (Dickerson and Kemeny, 2004). Indeed, we found no evidence for arousal-induced cortisol changes, *F*s < 1.

**Figure 3:**
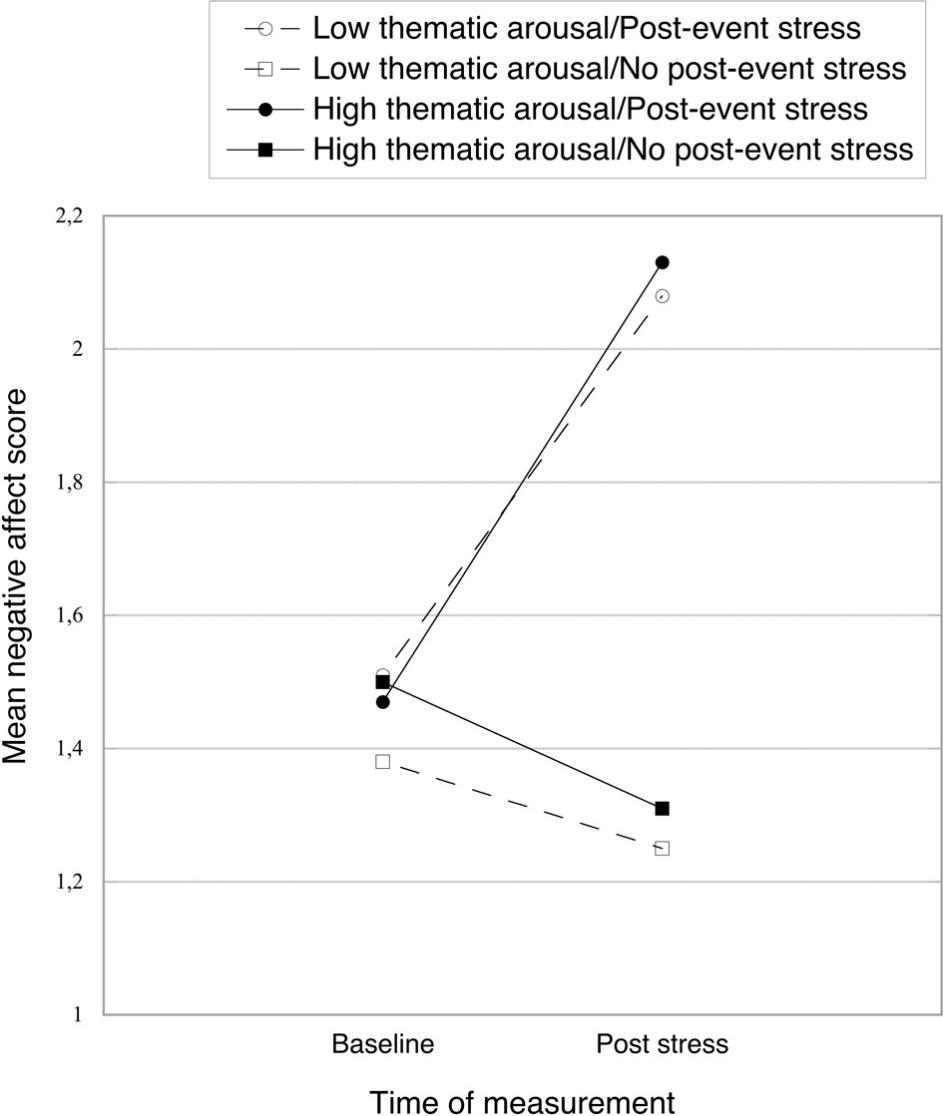
**Mean negative affect scores from PANAS (baseline and post-stress) as a function of thematic arousal and post-event stress.** Higher values indicate more negative affect.

In the stress condition participants' reported affect became more negative (see Figure 3), as indicated by a significant interaction between post-event stress and measurement time [*F*_(1, 84)_ = 53.52, *p* < 0.001, η^2^_p_ = 0.39] from a mixed 2 (post-event stress vs. no post-event stress) × 2 (low vs. high thematic arousal) × 2 (baseline vs. post-stress measure) ANOVA. This interaction qualified significant main effects of post-event stress [*F*_(1, 84)_ = 27.58, *p* < 0.001, η^2^_p_ = 0.25] and measurement time stress [*F*_(1, 84)_ = 19.05, *p* < 0.001, η^2^_p_ = 0.19]. Whereas negative affect did not differ between the stress group (*M* = 1.49, SD = 0.42) and the no-stress control group at baseline (*M* = 1.44, SD = 0.36) [*F* < 1, *ns*], it was significantly more negative after the stress induction (*M* = 2.10, SD = 0.67, vs. *M* = 1.28, SD = 0.27; for the stress and control group, respectively), *F*_(1, 84)_ = 55.10, *p* < 0.001, η^2^_p_ = 0.40. No other significant effects emerged, all *F*s < 1, *ns*.

### Memory for event items

Table 1 (left panel) contains the mean recognition rates for event items (proportions of hits, i.e., correct *yes*-responses). The data were entered into a mixed 2 (post-event stress vs. no post-event stress) × 2 (low vs. high thematic arousal) × 2 (central vs. peripheral items) × 2 (rehearsed vs. non-rehearsed item) ANOVA, with the first two variables varied between subjects and the latter two varied within subjects. Consistent with previous findings for the present eyewitness material (Echterhoff et al., 2007), the hit rate for central event items (*M* = 0.82, SE = 0.01) was higher than the hit rate for peripheral event items (*M* = 0.42, SE = 0.02), as indicated by a significant main effect of item centrality, *F*_(1, 84)_ = 317.51, *p* < 0.001, η^2^_p_ = 0.79. Also, the additional presentation of information in the post-event description enhanced recognition memory: the hit rate for rehearsed event items (*M* = 0.67, SE = 0.02) was higher than for non-rehearsed event items, i.e., event items not included in the post-event description (*M* = 0.57, SE = 0.02), as revealed by a significant main effect of rehearsal, *F*_(1, 84)_ = 26.84, *p* < 0.001, η^2^_p_ = 0.24. We found no significant interactions between rehearsal, on the one hand, and arousal and/or post-event stress, on the other hand, all *F*s < 1. Hence, it was not necessary to include rehearsal in the subsequent analyses of arousal and stress effects.

**Table 1:** **Mean recognition rates (standard deviations) as a function of thematic arousal, post-event stress, and item type**.

**Group**	**Event items**						**Non-event items**	
	**Peripheral/non-rehearsed**	**Peripheral/rehearsed**	**Peripheral/all**	**Central/non-rehearsed**	**Central/rehearsed**	**Central/all**	**False additional**	**New**
	***M* (SD)**	***M* (SD)**	***M* (SD)**	***M* (SD)**	***M* (SD)**	***M* (SD)**	***M* (SD)**	***M* (SD)**
**LOW THEMATIC AROUSAL**								
Post-event stress	0.39 (0.21)	0.53 (0.23)	0.46 (0.18)	0.79 (0.14)	0.88 (0.13)	0.83 (0.09)	0.52 (0.20)	0.32 (0.19)
No post-event stress	0.29 (0.24)	0.43 (0.30)	0.36 (0.24)	0.78 (0.15)	0.84 (0.16)	0.81 (0.13)	0.37 (0.26)	0.26 (0.15)
**HIGH THEMATIC AROUSAL**								
Post-event stress	0.33 (0.22)	0.42 (0.26)	0.37 (0.20)	0.83 (0.14)	0.89 (0.13)	0.86 (0.10)	0.42 (0.25)	0.28 (0.17)
No post-event stress	0.40 (0.24)	0.54 (0.24)	0.47 (0.18)	0.76 (0.18)	0.80 (0.14)	0.78 (0.13)	0.50 (0.21)	0.39 (0.20)

Note: The scores for peripheral/all and central/all are collapsed across the non-rehearsed and rehearsed data. See text for further explanations of item types.

Importantly, the ANOVA also yielded the predicted significant post-event stress × thematic arousal × centrality interaction, *F*_(1, 84)_ = 7.26, *p* = 0.009, η^2^_p_ = 0.08. (The ANOVA yielded no other significant effects, all *F*s < 1.84, all *p*s > 0.18.) This three-way interaction supports the notion that the level of memory enhancement for central (vs. peripheral) event items depends on the interaction of thematic arousal and post-event stress. We note that this interaction remained significant in separate analyses for both rehearsed and non-rehearsed event items, *F*_(1, 84)_ = 5.12, *p* = 0.026, η^2^_p_ = 0.06, and *F*_(1, 84)_ = 4.31, *p* = 0.041, η^2^_p_ = 0.05, respectively.

**Figure 4:**
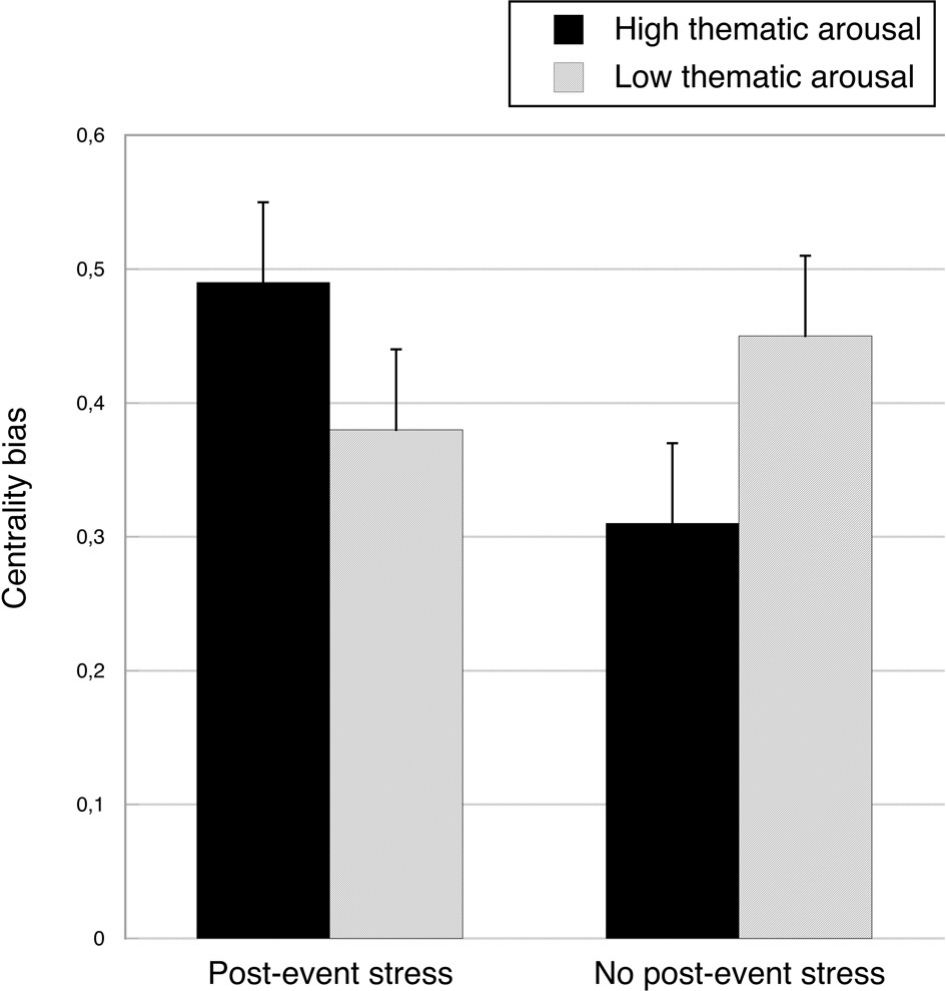
**Mean centrality bias (hit rate for central event items—hit rate for peripheral event items) as a function of post-event stress and thematic arousal, collapsed across rehearsal conditions.** Bars represent standard errors.

A closer examination of the interaction showed that, consistent with our prediction, the recognition advantage of central event items over peripheral event items was most pronounced under high thematic arousal and post-event stress (see the recognition rates for periperhal/all and central/all event items in Table 1). We used a complex, weighted contrast to test whether this difference was significant, coding the high thematic arousal/post-event-stress condition with +1, and each of the other three conditions with –1/3. For ease of interpretation, we calculated a *centrality bias* by subtracting the hit rate for peripheral event items from the hit rate for central event items, with greater values indicating a stronger centrality bias. The means and standard errors for the four experimental groups are depicted in Figure 4. Importantly, the mean centrality bias in the high thematic arousal/post-event-stress group (*MD* = 0.49, SD = 0.21) was significantly greater than the mean centrality bias in the three other conditions (*MD* = 0.38, SD = 0.22), *F*_(1, 84)_ = 4.41, *p* = 0.039, η^2^_p_ = 0.05. According to Cohen (1988), both the critical interaction and the latter contrast yielded medium-size effects.

There was no significant difference between the low thematic arousal/no-post-event-stress group and the low thematic arousal/post-event-stress group, *F* = 1.19, *ns*. When no post-event stress was induced, the centrality bias was even greater in the low thematic arousal condition than in the high thematic arousal condition, *F*_(1, 84)_ = 4.32, *p* = 0.041, η^2^_p_ = 0.05.

We also explored possible differences between the four arousal/post-event stress groups *separately* for central and peripheral details. The hit rate for central event items was significantly higher under thematic arousal plus post-event stress (*M* = 0.86) than under thematic arousal without post-event stress (*M* = 0.78), *F*_(1, 84)_ = 5.17, *p* = 0.026, η^2^_p_ = 0.06. This effect is consistent with the idea that additional post-event stress after arousal enhances memory for central information.

No other difference reached conventional significance levels; for central event items, *F*s < 2.13, *p*s > 0.15. For peripheral event items we found the following trends: there was a trend toward a lower hit rate in the high thematic arousal plus post-event stress group (*M* = 0.37) than in the high thematic arousal/no post-event stress group (*M* = 0.47), *F*_(1, 84)_ = 2.60, *p* = 0.111, η^2^_p_ = 0.03, and toward a higher hit rate in the latter group compared to the low thematic arousal/no-post-event stress group (*M* = 0.36), *F*_(1, 84)_ = 3.19, *p* = 0.078, η^2^_p_ = 0.04. Hence, in the absence of stress, thematic arousal did not reduce but, if anything, even enhanced the hit rate for peripheral event items. There was also a trend toward a lower hit rate for peripheral items in the no-post-event stress/low thematic arousal group (*M* = 0.36) compared to the post-event stress/low thematic arousal group (*M* = 0.46), *F*_(1, 84)_ = 2.54, *p* = 0.115, η^2^_p_ = 0.03.

### Memory for non-event items

Table 1 (right panel) contains the mean recognition rates for non-event items, separately for items falsely added in the post-event description and completely new items. Overall, the rate of erroneous *yes*-responses for false additional items was significantly higher than the rate of erroneous *yes*-responses for completely new items [*F*_(1, 84)_ = 36.69, *p* < 0.001, η^2^_p_ = 0.30], as calculated with a mixed 2 (post-event stress vs. no post-event stress) × 2 (low vs. high thematic arousal) × 2 (false additional vs. new) ANOVA. This effect captures the well-known effect of post-event misinformation (Loftus, 2005). The effect did not differ across the experimental groups, all *F*s < 1.24, *ns*. Thus, there was no evidence that stress and/or thematic arousal moderated the influence of falsely suggested misinformation.

### Response bias

We also examined if the key finding (i.e., the interaction between thematic arousal and post-event stress for the acceptance of central vs. peripheral event items) could possibly be due to differences participants' response bias. We calculated a standard response bias estimate (*B*_*r*_) following the formula proposed by Snodgrass and Corwin (1988), FA/[1 − (H − FA)], with FA representing the false alarm rate (accepted new items) and H the hit rate for all event items (central and peripheral). The 2 (post-event stress vs. no post-event stress) × 2 (low vs. high thematic arousal) ANOVA yielded no significant interaction effect, *F*_(1, 84)_ = 2.10, *p* = 0.16. When we included the hit rate only for central items and only for peripheral items in the calculation of the response bias, the interaction effects remained non-significant, both *F*s < 1, *p*s > 0.44. Hence, there was no evidence that response bias differences contributed to our main finding.

### Mediation of the centrality effect of post-event stress under high thematic arousal

The previous analyses revealed that participants experiencing post-event stress under high thematic arousal exhibited a greater memory centrality bias (a positive difference between the recognition rate for central event items and the recognition rate for peripheral event items) than participants experiencing only high thematic or post-event-stress. Finally, we examined whether this centrality effect of post-event stress under high thematic arousal condition was statistically mediated by the cortisol response, our main biological stress marker.

**Figure 5:**
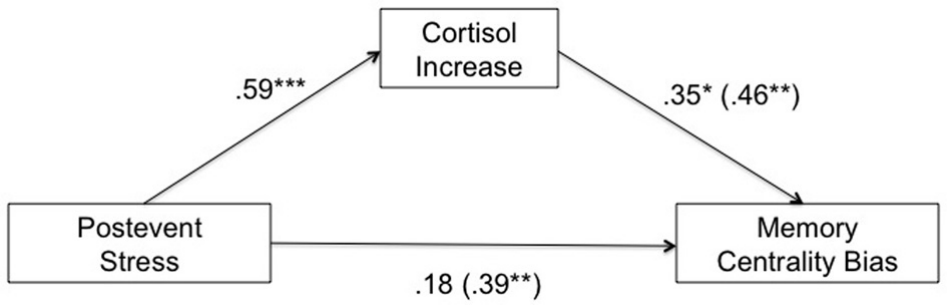
**Mediation analysis for the high thematic arousal condition, with post-event stress (control = −1, stress = +1) as independent variable, cortisol increase as mediator, and memory centrality bias (proportion of accepted central event items minus proportion of accepted peripheral event items) as dependent variable.** Path coefficients are standardized β-coefficients from (multiple) regression analyses. The numbers in parentheses represent the direct effect (bivariate β-coefficients) of each of the two predictors (post-event stress and cortisol increase) prior to the inclusion of the other predictor. See text for the calculation of cortisol increase. ^*^p < 0.05, ^**^p < 0.01, ^***^p < 0.001 (two-tailed).

As recommended by Baron and Kenny (1986) we conducted bivariate regressions from post-event stress on cortisol increase and from cortisol increase on centrality bias and a stepwise linear regression for the memory centrality bias as dependent variable, with post-event stress as the single independent variable entered in Step 1, and the proposed mediator, cortisol increase, as an additional independent variable entered in Step 2. The main findings from these regressions are summarized in Figure 5. We found that all four standard conditions of mediation proposed by Baron and Kenny (1986) were met. Consistent with the above ANOVAs, both the centrality bias and cortisol increase were significantly higher in the post-event-stress condition than the no-stress condition, β = 0.39, *t*_(43)_ = 2.75, *p* = 0.009, and β = 0.59, *t*_(43)_ = 4.77, *p* < 0.001 (Conditions 1 and 2, respectively). Also, larger cortisol increase was associated with a higher centrality bias, β = 0.46, *t*_(43)_ = 3.34, *p* = 0.002 (Condition 3). When post-event stress (contrast-coded: without stress = –1, with stress = +1) and cortisol increase were both included as predictors of centrality bias, only cortisol increase remained significant, β = 0.35, *t*_(42)_ = 2.07, *p* = 0.045, whereas the effect of post-event stress was reduced to non-significance, β = 0.18, *t*_(42)_ = 1.09, *p* = 0.283 (Condition 4). The indirect effect of post-event stress on the centrality bias via cortisol increase was significant in a Sobel test of mediation (Sobel, 1982), *Z* = 1.86, *p* = 0.031 (one-tailed). These findings show that the effect of post-event stress on the centrality bias under high thematic arousal was mediated by the cortisol response.

## Discussion

In our study, eyewitnesses' memory for a witnessed event was influenced by the combined effect of thematic arousal during encoding and subsequent social stress, which was unrelated to the event itself. The affected dimension was the centrality bias in recognition memory, that is, the recognition advantage for central event items over peripheral event items. This centrality bias was more pronounced under high thematic arousal and post-event stress than under high thematic arousal without post-event stress or under post-event stress without thematic arousal. In other words, the centrality bias in memory was greater when thematic arousal during the witnessing of the incident was followed by stress compared to the conditions in which participants experienced either arousal or post-event stress. The effect on the centrality bias was apparently due to both an increased hit rate for central event items and a decreased hit rate for peripheral event items. Thus, although we did not find effects on the overall quantity of remembered information, we could demonstrate differences in the type or quality of remembered information (central vs. peripheral).

This finding is consistent with previous research that found an interaction between arousal during encoding and post-encoding stress (Cahill et al., 2003). However, our study remedies a weakness of this extant work, that is, the potential confounds of the arousal manipulation. Inevitably, arousing stimulus material differs from non-arousing material in ways that are unrelated to arousal or emotional valence, for instance, unexpectedness, visual salience, or relatedness to other knowledge. In contrast, by manipulating the perception of the material with identical stimulus material, we avoided such potential confounds (also see, e.g., Laney et al., 2004; Payne et al., 2007). Furthermore, we distinguished between peripheral and central stimulus information. The design of our study allowed us to detect opposite effects of arousal followed by post-event stress for peripheral and central event items. We could thus demonstrate previously unknown conditions for the narrowing of memory, which has been a key concept of research on the interplay of emotion and cognition (see Christianson, 1992).

Our results have implications for legal and criminological practice, particularly the treatment of eyewitnesses. Legally relevant events like accidents and crimes are likely to induce arousal in common eyewitnesses. Hence, eyewitnesses are likely to have experienced arousal during observation of an incident. They may also be exposed to social stress shortly after the incident, for instance, during an interrogation. Our study suggests that legal practitioners should be aware that such a combination of arousal during observation and post-observation stress could lead to a focus on central aspects at the expense of peripheral details in eyewitnesses' subsequent memory. This would be particularly undesirable when information about peripheral elements of an incident, for example, details indicating the use of a tool or weapon, is relevant to the investigation (see Osterburg, 2010).

To our knowledge, our study is the first to examine the role of neuroendocrine processes in stress effects on eyewitness memory, which is a key domain of applied cognitive psychology. We obtained evidence for the neuroendocrine mechanism underlying the observed centrality bias in eyewitness memory by means of a mediation analysis. Since post-event stress led to a centrality bias only under high thematic arousal, we restricted the analysis for the stress effect to the high-arousal condition. We found that—under high thematic arousal—the stress effect was statistically mediated by cortisol increase. This finding is consistent with the notion that cortisol increase is a key biopsychological process driving the effect of social stress on memory (Wolf, 2009).

Taken together, the findings in the stress condition are in line with the idea that an interaction of the SNS and the HPA axis boosts emotional memory via their joint effects on the amygdala (Cahill and McGaugh, 1998; Roozendaal et al., 2009). The enhanced memory for the central, or gist, information, which resulted from the interaction of thematic arousal and stress, is consistent with neuropsychological evidence of a special role of the amygdala in memory for the gist of emotional events (Adolphs et al., 2005).

In the current study we did not obtain a physiological measure of arousal during the presentation of the video. Future studies could assess heart rate, electrodermal activity, the startle reflex, or the enzyme salivary alpha amylase. All these measures have been shown to be responsive to the presentation of emotional arousing material (Lang et al., 1990, 1993; Segal and Cahill, 2009). Moreover, previous work suggests that when these measures are obtained during stress, they can predict, in combination with cortisol measures, emotional long-term memory (Smeets et al., 2008; Zoladz et al., 2011).

We found no evidence that thematic arousal and/or post-event stress affected participants' memory for false post-event information. Given the extant evidence, this result should not be taken as a surprise. For other types of false memory the findings are inconsistent: Some researchers have found effects (Payne et al., 2002), whereas others have not (Smeets et al., 2006, 2008). Also, the scant evidence in the domain of memory suggestibility has not revealed any stress effects (Eisen et al., 2002).

Apart from a few false details, the post-event narrative correctly described the events in the eyewitness video, which provided participants with an opportunity to rehearse the original stimulus material. As expected, rehearsal (that is, inclusion vs. omission in the post-event narrative) enhanced the correct recognition rate. We note that the presentation of the post-event narrative fell into the phase of pronounced cortisol response to the previous stress induction. However, rehearsal of event items was not found to interact with post-event stress (and neither with thematic arousal). Hence, there was no evidence that post-event stress moderated the rehearsal effects.

Interestingly, when post-event stress was absent, the centrality bias was lower under high (vs. low) thematic arousal. Also, when arousal was low, the centrality bias was relatively small under post-event stress. Thus, there was no evidence for memory narrowing when arousal or post-event stress occurred alone. As the data presented in Figure 4 suggest, the centrality bias found in the baseline condition (low arousal, no stress) apparently decreased when either arousal or post-event stress were added, but then increased again when arousal and stress were combined. The exploratory data analyses suggest that these differences in the centrality bias were predominantly due to differences in the memory for peripheral event items: In the absence of post-event stress, thematic arousal did not lead to a reduction but, if anything, an increase in the hit rate for peripheral event items. In the low-thematic arousal group, post-event stress did not lead to a decrease but, if anything, a rise in the hit rate for peripheral event items.

While we have no data that directly speak to this issue, we think it is stimulating to consider possible factors that could account for this interesting pattern. According to the arousal-biased competition model (Mather and Sutherland, 2011), arousal enhances the priority of goal-relevant information over goal-irrelevant information (for an application to eyewitness memory, see Hope et al., 2012). This approach could explain the findings in the stress/low-arousal and no-stress/high-arousal groups if one can make a convincing case why peripheral details were perceived as more relevant than central details. For instance, given the nature of our stimulus materials our student participants might have believed that details of the incident were particularly important. However, we have no evidence that bears directly on this claim. Also, a challenge for this approach is to explain the enhanced centrality bias in the arousal-plus-post-event-stress condition.

According to other research, thematic arousal can enhance memory for all aspects, including peripheral elements, of an observed event (Laney et al., 2004; also see Libkuman et al., 1999). This account could explain the enhanced memory for peripheral details in the high arousal/no-stress group. Indeed, thematic arousal, specifically apprehension about an upsetting turn of events, could induce participants to explore the witnessed material in more detail. However, it remains unclear how this account could cover the effects of post-event stress and the increased centrality bias in the arousal-plus-post-event-stress condition.

Another perspective is offered by research on mood effects on information processing. According to the mood-as-information approach (see Schwarz and Clore, 1996; Bless and Fiedler, 2006), positive and pleasant mood states inform the perceiver that the current situation poses no problems or risks, whereas negative and unpleasant mood states signal potential problems or threats in the current situation. While positive mood allows global, gist-oriented processing, negative mood induces the perceiver to engage in detail-oriented processing, which is typically adaptive in problem handling. By this view, the hit rate for peripheral details under arousal or post-event stress might be due to an increase in detail-oriented information processing that is triggered by the corresponding unpleasant mood state. A difficulty faced by this view is the lack of increased negative affect in the no stress/high arousal condition in our study (see Figure 3). However, the affect measure was administered approximately 15 min after the arousal manipulation, which may have been too late to detect existing mood differences.

A mood-as-information account would have to be supplemented by an explanation for the reduced memory for peripheral details under both arousal and post-event stress: such an explanation would have to assume the existence of a critical threshold of activation or arousal, at which gist- or priority-oriented information processing (Mather and Sutherland, 2011) takes over and subdues or prevents detailed-oriented processing. In our study, this threshold might be reached when high arousal is immediately followed by the stress induction. By this view, the effect of arousal on information processing, specifically memory for central vs. peripheral details, approximates an inverted U-shape function (for similar concepts, see Abercrombie et al., 2003; Rimmele et al., 2003; Diamond et al., 2007). A moderate increase in arousal induces detail-oriented processing, whereas a stronger increase reduces detail-oriented processing in favor of a focus on central or globally relevant stimuli. At the neuroendocrine level, a strong increase would be reflected by a joint activation of the SNS and the HPA.

In sum, our experiment demonstrates that thematically induced arousal and post learning stress interact in a complex fashion to enhance the centrality bias in recognition memory. Because we used thematic arousal, the results cannot reflect differences in stimulus quality. Moreover, since stress was administered after learning we can exclude effects on attention or initial encoding. The mediation analysis revealed that the increased centrality bias was mediated by the stress induced cortisol increase. These findings extend previous observations (e.g., Cahill et al., 2003) and suggest that the interaction of noradrenergic arousal with the stress hormone cortisol, most likely via joint effects on the amygdala, enhances emotional memories for central details.

### Conflict of interest statement

The authors declare that the research was conducted in the absence of any commercial or financial relationships that could be construed as a potential conflict of interest.
